# Nanotechnology and nanomaterials in the treatment and diagnosis of cancer: Correspondence

**DOI:** 10.1097/MS9.0000000000000677

**Published:** 2023-04-18

**Authors:** Soumeia Zeghoud, Deepak Chandran, Ilham Ben Amor, Hadia Hemmami, Pran Mohankumar, Talha Bin Emran

**Affiliations:** a Department of Process Engineering and Petrochemical, Faculty of Technology; b Renewable Energy Development unit in Arid Zones (UDERZA), University of El Oued, El Oued, Algeria; c Department of Veterinary Sciences and Animal Husbandry, Amrita School of Agricultural Sciences, Amrita Vishwa Vidyapeetham University; d School of Agricultural Sciences, Karunya Institute of Technology and Sciences, Coimbatore, Tamil Nadu, India; e Department of Pharmacy, BGC Trust University Bangladesh, Chittagong; f Department of Pharmacy, Faculty of Allied Health Sciences, Daffodil International University, Dhaka, Bangladesh

*Dear Editor*,

The key to winning the fight against cancer is early cancer diagnosis. The imaging methods that are frequently used to diagnose cancer include X-rays, ultrasonography, computed tomography scans, MRI, and PET scans. Final confirmation of cancer is aided by morphological alterations in tissues or cells (through cytology or histopathology). Some methods only identify cancer after tissue alterations become apparent, by which time the disease may have spread and resulted in metastasis. The inability of traditional imaging methods to differentiate between benign and malignant tumours is another drawback. Moreover, cytology and histopathology are not reliable, independent methods for early cancer detection. Innovative molecular contrast media and materials made possible by nanotechnology allow for more rapid and precise initial diagnosis as well as continuing evaluation of cancer patient treatment^1^.

Although they have not yet been utilised to actually diagnose cancer, nanoparticles are now being used in a number of medical screening procedures. One of the materials most frequently seen in at-home test strips is gold nanoparticles. The fact that nanoparticles have a higher surface area to volume ratio than their bigger counterparts makes them an excellent choice for the detection of cancer. Due to this characteristic, the nanoparticle is densely coated with molecules such as polyethylene glycol, aptamers, fluorescent probes, small molecules, and antibodies. This boosts the selectivity and sensitivity of the bioassay by presenting several binding ligands for cancer cells (multivalent impact of nanotools)^2^. Nanotechnology is used in diagnostics for in-vivo imaging and the identification of extracellular cancer biomarkers. A good nanoprobe has to circulate for a long period, be selective for cancer cells, and not be hazardous to adjacent tissue^3^,^4^.

Nanodevices have been investigated for the detection of surrounding healthy tissues’ toxicity and blood indicators. Cancer-related circulating tumour cells, related proteins or cell surface proteins, carbohydrates or circulating tumour nucleic acids, and tumour-shed exosomes are some examples of these indicators. Although it is generally recognised that these biomarkers aid in the early detection of cancer, they also aid in the monitoring of treatment and recurrence^5^. They have drawbacks include low body fluid concentrations, variability in amounts and timings between individuals, and challenging prospective investigations. Nanotechnology, which provides great specificity and sensitivity, overcomes these obstacles. Using nano-enabled sensors, multiplexed measurements, high sensitivity, and specificity are all feasible. Next-generation devices combine capture with genetic analysis to better clarify an issue^6^,^7^.

Nanotechnology makes use of nanoprobes that may be targeted passively or actively to accumulate specifically in tumour cells. The difficulties include the targeting of tumours, the interaction of nanoparticles with blood proteins, and their removal by the reticuloendothelial system. Due to extravasation from blood arteries, passive targeting implies a bias for collecting the nanoparticles in solid tumours. This is made feasible by the tumour’s faulty angiogenesis, in which the new blood vessels lack tight connections in their endothelial cells, allowing nanoparticles as small as 150 nm to seep out and accumulate preferentially in the tumour tissue. Enhanced permeability and retention is the name given to this phenomena (EPR). Active targeting entails the tumour cell surface receptors recognising nanoparticles. This will improve the in-vivo tumour detection method’s sensitivity. Active targeting will produce better outcomes than passive targeting for early cancer diagnosis^8^,^9^.

Nanoparticles can be used for gene therapy, radiation, immunotherapy, and delivery of chemotherapy. Delivery of chemotherapy aims to reduce drug toxicity and improve pharmacokinetics by selectively targeting and delivering chemotherapy to cancer tissues. This mainly relies on passive targeting, which makes use of the EPR effect previously discussed^8^,^10^. Drugs have a longer half-life thanks to nanocarriers. Based on a knowledge of the interplay between the tumour and host, immunotherapy is a potential new approach to treating cancer. The delivery of immunostimulatory or immunomodulatory substances via nanotechnology is being researched. It can be used in conjunction with other treatments^11^,^12^.

About radiotherapy This nanotechnology technology has applications in the targeted delivery of radioisotopes and radiosensitizers, reduced side effects of radiotherapy by reducing distribution to healthy tissues, combining radiotherapy with chemotherapy to achieve synergism but prevent side effects, and delivering image-guided radiotherapy^5^,^13^. Regarding gene therapy, although there is a great deal of interest in the field, the results are still too preliminary for clinical use. Finding a mechanism to achieve these effects is difficult despite a large range of medicines focused at gene regulation, such as gene silencing, RNA interference, anti-sense therapy, and gene and genome editing. The use of nanoparticles as gene therapy carriers has several benefits, including simple design and functionalization, minimal immunogenicity, and low toxicity. Future applications for gene-targeted delivery utilising nanoparticles are particularly promising. Although it is still in its early stages, gene therapy is extremely promising^14^,^15^.

Moreover, from Nanodelivery Systems The quantum dot Quantum dots (QDs), which are semiconductor nanocrystals, exhibit exceptional physical characteristics. QDs-based probes have made significant advancements in in-vivo and cellular molecular imaging. Growing research is indicating that technology based on QDs may become an attractive method in cancer research^16^. Biocompatible QDs for in-vitro cancer cell mapping were first presented in 1998. They were used by scientists to create QD-based cancer imaging probes that were coupled to ligands, antibodies, or peptides that are unique to the disease. Compared with conventional immunohistochemistry, QD-immunohistochemistry is more sensitive and specific and can evaluate even low levels, providing significantly more information for tailored therapy. QD imaging has become a promising tool for cancer early detection^17^. An outstanding example of how fusing nanoscience and biomedicine might resolve a biological issue is nanoshells and AuNPs/gold nanoshells (AuNSs). For the best tissue penetration, their tunable surface plasmon resonance may be tuned to the near-infrared. The extraordinarily efficient light-to-heat transfer of AuNSs causes thermal destruction of the tumour during laser irradiation without causing damage to healthy tissues. And Dendrimers: AuNSs can even be utilised as a carrier for a variety of medicinal and diagnostic substances^18^. They are unique nanoarchitectures with distinctive features such a monodispersed uni-micellar nature, a nanometric size range, and a spherical three-dimensional form. Dendrimers’ biocompatibility has been used to administer potent drugs like doxorubicin. By affixing ligands to the surfaces of cancerous cells, this nanostructure targets those cells. For the purpose of providing contrast chemicals for magnetic resonance imaging and cancer therapies, dendrimers have undergone extensive research. Their toxicity was greatly lowered by the surface’s gold coating, although their size was little affected. Moreover, it acted as an anchor to secure compounds with high affinity for targeting to tumour cells^19^. Nanoparticles that are liposomal They play a part in delivery to a particular target site, reduce biodistribution toxicity due to the surface-modifiable lipid content, and resemble cell membranes in terms of structure. Targeting certain cancer cells is a benefit of liposome-based theranostics (particles created to deliver medicinal and diagnostic molecules at the same time). Liposomes boost the drug’s solubility and make them more stable in the circulation. They also serve as preparations for prolonged release and shield the medication from deterioration and pH changes, extending the drug’s half-life in circulation. Multidrug resistance is overcome with the use of liposomes. Liposome administration is employed with medications such doxorubicin, daunorubicin, mitoxantrone, paclitaxel, cytarabine, and irinotecan^20^,^21^.

One of the most significant scientific breakthroughs in recent years has been the development of nanotechnology, which has not only transformed the engineering sector but is also having an influence on the medical and paramedical sectors. The qualities and characteristics of these nanomaterials have been successfully identified by scientists, who have then optimised them for usage in the healthcare sector. Although if certain nanoparticles have not been successful in the clinic, other innovative and fascinating nanoparticles are now being studied and have tremendous promise, suggesting that new therapeutic options may soon be accessible. Nanomaterials are incredibly adaptable and have a number of advantages that can improve cancer treatments and diagnostics^22^.

They are very helpful as drug delivery vehicles because of their small size and distinct binding characteristics. In contemporary clinical procedures, daunorubicin, medications including doxorubicin, mitoxantrone, irinotecan, cytarabine paclitaxel, and amphotericin B are conjugated with liposomes for their distribution. Key elements of cancer chemotherapy include doxorubicin, vincristine, cytarabine, daunorubicin, paclitaxel, and mitoxantrone. Even in cancer diagnostics, particles like dendrimers, nanoshells, and gold nanoparticles are presently used for imaging and tumour marker detection^23^.

The production costs, extensibility, complexity, health safety, and possible toxicity of this unique technology are its drawbacks. Extensive study and clinical trials have successfully addressed these, and nanomedicine is now one of the major global enterprises. Because to breakthroughs in nanomedicine, a valuable assortment of research tools and clinically beneficial gadgets will soon be made available. In their new commercial applications, pharmaceutical firms will make advantage of in-vivo imaging, cutting-edge treatments, and improved drug delivery technology. When employed to treat brain tumours, neuro-electronic interfaces and cell healing technologies might revolutionise medicine and the healthcare sector^24^.

## Ethical approval

Nil.

## Consent

None declared.

## Source of funding

This compilation is a correspondence article written by its authors and required no substantial funding to be stated.

## Author contribution

S.Z.: conceptualization, data curation, writing—original draft preparation, writing—reviewing and editing. D.C.: data curation, writing—original draft preparation, writing—reviewing and editing. I.B.A., H.H.: writing—original draft preparation, writing—reviewing and editing. P.M., T.B.E.: writing—reviewing and editing, visualisation, supervision.

## Conflicts of interest disclosure

All authors declare that there exist no commercial or financial relationships that could, in any way, lead to a potential conflict of interest.

## Provenance and peer review

Not commissioned, externally peer-reviewed.

## Acknowledgements

All the authors acknowledge and thank their respective Institutes and Universities.
